# Coronavirus disease 19 in minority populations of Newark, New Jersey

**DOI:** 10.1186/s12939-020-01208-1

**Published:** 2020-06-10

**Authors:** Alexis K. Okoh, Christoph Sossou, Neha S. Dangayach, Sherin Meledathu, Oluwakemi Phillips, Corinne Raczek, Michael Patti, Nathan Kang, Sameer A. Hirji, Charles Cathcart, Christian Engell, Marc Cohen, Sandhya Nagarakanti, Eliahu Bishburg, Harpreet S. Grewal

**Affiliations:** Heart and Lung Research Center, RWJ Barnabas Health, Newark Beth Israel Medical Center, 201 Lyons, Avenue, Suite G5., Newark, New Jersey 07112 USA

**Keywords:** COVID-19, African American, Minority, Hispanic, mortality, morbidity

## Abstract

**Background:**

The purpose of this study is to report the clinical features and outcomes of Black/African American (AA) and Latino Hispanic patients with Coronavirus disease 2019 (COVID-19) hospitalized in an inter-city hospital in the state of New Jersey.

**Methods:**

This is a retrospective cohort study of AA and Latino Hispanic patients with COVID-19 admitted to a 665-bed quaternary care, teaching hospital located in Newark, New Jersey. The study included patients who had completed hospitalization between March 10, 2020, and April 10, 2020. We reviewed demographics, socioeconomic variables and incidence of in-hospital mortality and morbidity. Logistic regression was used to identify predictor of in-hospital death.

**Results:**

Out of 416 patients, 251 (60%) had completed hospitalization as of April 10, 2020. The incidence of In-hospital mortality was 38.6% (n = 97). Most common symptoms at initial presentation were dyspnea 39% (n = 162) followed by cough 38%(n = 156) and fever 34% (n = 143). Patients were in the highest quartile for population’s density, number of housing units and disproportionately fell into the lowest median income quartile for the state of New Jersey. The incidence of septic shock, acute kidney injury (AKI) requiring hemodialysis and admission to an intensive care unit (ICU) was 24% (n = 59), 21% (n = 52), 33% (n = 82) respectively. Independent predictors of in-hospital mortality were older age, lower serum Hemoglobin < 10 mg/dl, elevated serum Ferritin and Creatinine phosphokinase levels > 1200 U/L and > 1000 U/L.

**Conclusions:**

Findings from an inter-city hospital’s experience with COVID-19 among underserved minority populations showed that, more than one of every three patients were at risk for in-hospital death or morbidity. Older age and elevated inflammatory markers at presentation were associated with in-hospital death.

## Background

Since the first case of the novel coronavirus disease 2019 (COVID-19) was reported in Wuhan, China, more than 2.7 million cases have been confirmed globally [1, 2]. Early studies have shown that, elderly patients with multiple co-existing comorbidities are more likely to have worse outcomes after COVID-19 [3]. The COVID-19 pandemic has affected over 180 countries and has had a drastic impact on all racial and socioeconomic segments of the population. However, it appears that COVID-19 has had an especially detrimental effect on vulnerable subjects and ethnic minorities. Staggering statistics from healthcare departments across the Unites States show that, more than two-thirds of COVID-19 deaths occurred in black individuals although, blacks consisted of less than a third of the population [4–6]. Underserved minority populations therefore stand to suffer disproportionately both from the pandemic and its aftermath mainly because they have limited access to healthcare, reside in densely populated areas, earn significantly lower income, or have less employment opportunities.

As the science surrounding this novel disease evolves, a greater attention must be paid to identifying individuals who suffer the most devastating effects. While recent studies have focused on describing the clinical disease course, outcomes and identifying modifiable risk factors, little is known about the effects of COVID-19 on minority populations in the United States.

Newark is the second-most racially diverse city in New Jersey and houses more than 50% Black/African American and 33% Hispanic or Latino population. Here, we present the clinical characteristics and outcomes in Black/African American and Latino/Hispanic COVID-19 patients who were managed at a quaternary care hospital in Newark, New Jersey.

## Methods

Setting, Population and Data Collection:

This study is a retrospective cohort study conducted at the Newark Beth Israel Medical Center (NBIMC), a 665-bed quaternary care, teaching hospital located in Newark, New Jersey. We reviewed data from a prospectively maintained COVID-19 database, which included all patients who had laboratory-confirmed severe acute respiratory syndrome coronavirus 2 (SARS-CoV-2) infection. This was defined as a positive result on a reverse-transcriptase-polymerase reaction (RT-PCR) assay of a specimen collected on a nasopharyngeal swab.

We included adults 18-years of age or older who were admitted between March 10, 2020 and April 10, 2020, with follow-up through April 20, 2020. Since most of the patient population served by NBIMC are Black/African Americans (AA) and Latino/Hispanics, non-AA or Latino/Hispanics were excluded from the study. Patients with full clinical data who had completed their hospitalization consisted the final study cohort. We compared outcomes between survivors vs. non-survivors.

The NBIMC institutional review board approved this study and waived the need for an informed consent due to its retrospective nature. We extracted de-identified data, on epidemiological, demographic, clinical, laboratory, treatment, and outcomes from electronic Medical records by using a standardized data collection protocol.

### Study definitions

We extracted data on co-morbid conditions by interrogating physician documentation and confirming with their international classification of diseases (ICD-9/10) codes. Presenting symptoms were subjectively classified according to patients complains at initial presentation.

An independent researcher (CS) who adjudicated any difference in interpretations between two senior author reviewers (AO, HSG) crosschecked collected data.

Other variables included population densities, housing units and median income representing the social determinants of health status among admitted patients. These variables were estimated by matching data from the United States Census Bureau [7], with 5-year estimates based on the residence zip codes of patients.

We defined sepsis and septic shock based on the 2016 Third International Consensus Definition for sepsis and septic shock [8]. We included in the definition of sepsis, the presence of any secondary infection as confirmed by a positive blood or urine culture or a new pathogen isolated from either the upper or lower respiratory tract. Acute respiratory distress syndrome (ARDS) was defined as acute onset hypoxemia (the ratio of the partial pressure of arterial oxygen to the fraction of inspired oxygen [Pao2:Fio2], < 300) with bilateral pulmonary opacities on chest imaging that were not fully explained by congestive heart failure or other forms of volume overload [9]. Acute kidney injury was defined according to the KDIGO clinical practice guidelines. We compared baseline characteristics and clinical outcomes between survivors and non-survivors.

### Statistical analysis

We summarized the data by using descriptive statistics, presenting continuous variables as median, interquartile range and categorical variables as proportions or percentages. We used the Mann- Whitney U test, Chi-square, or Fisher’s exact tests to compare differences between survivors and non-survivors.

Univariate and multivariable logistic regression models were used to explore the risk factors associated with in-hospital mortality. We performed a stepwise regression model with backward selection using the minimum Akaike Information criterion to identify independent predictors of in-hospital mortality. To avoid overfitting, we tested for variables that were significantly associated with in-hospital mortality at a univariate level and only included them in the final model if they had a p value of < 0.05.

Variables were not included in the univariate analysis if (a) their accuracy were not confirmed, such as self-reported presenting symptoms; (b) they showed collinearity or (c) their event rate was too low to calculate odds ratios. For laboratory values, we used sensitivity analysis to determine cut-offs based on their association with in-hospital death. Cut-offs were determined based on both clinical context and a sensitivity ≥80% in predicting in-hospital death. Statistical significance was considered when a two-sided alpha of less than 0.05 was reached. All analyses were performed using the JMP version 14.0.2 statistical software (SAS Institute Inc., Cary, NC, USA).

## Results

Between March 10, 2020 and April 10, 2020, we identified 416 COVID-19 adult patients who had at least a follow up through April 20, 2020. We excluded 35 (8.4%) patients who were non-AA or Latino/Hispanics. Of the excluded patients, only 10 had completed hospitalization at the time of study analysis. As of April 20, there were 381 patients who had completed hospitalization. Among these, 130 were still hospitalized and 251 had either died or been discharged. In-hospital mortality was recorded in 97 (38.6%) patients.

**Fig. 1:**
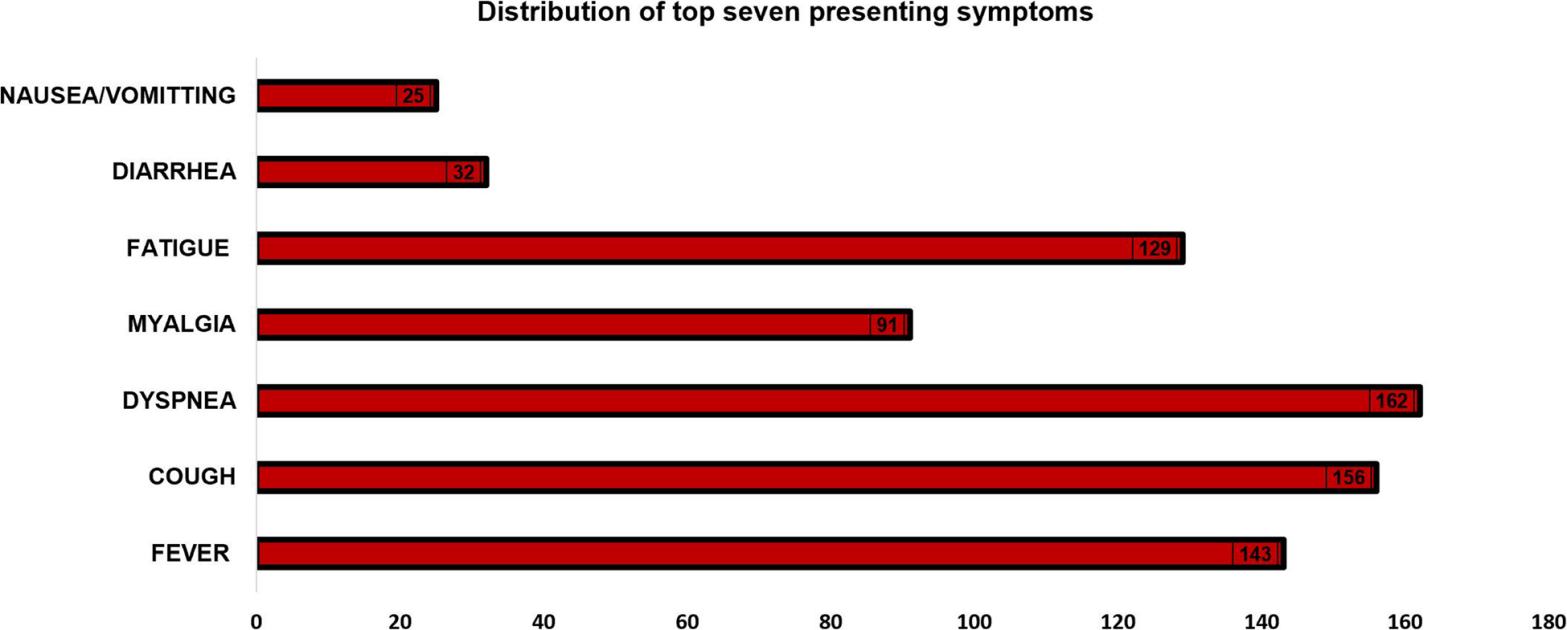
Distribution of common presenting symptoms of underserved minority patients admitted to an inter-city hospital in Newark, New Jersey for COVID-19

**Fig. 2:**
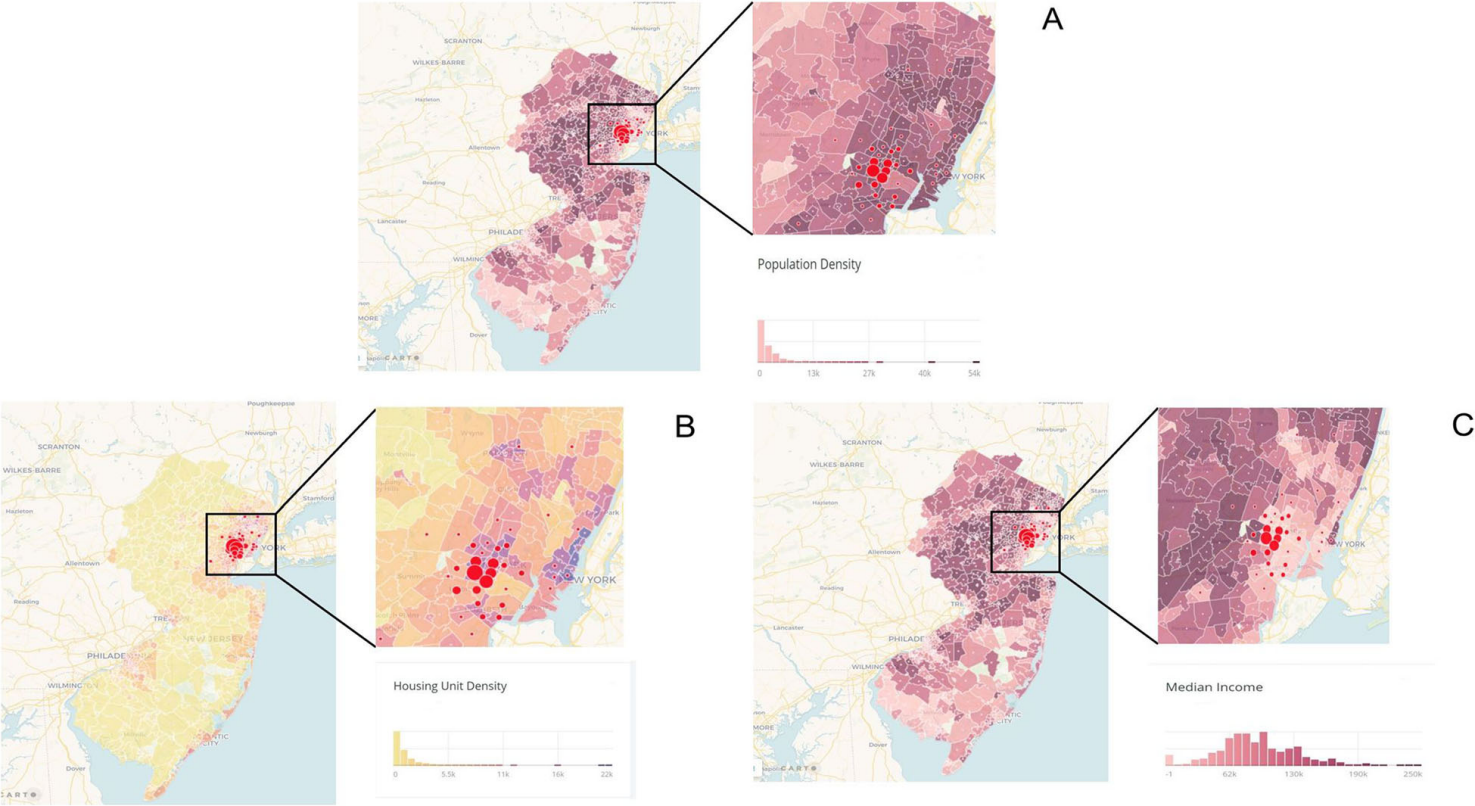
Heat map representation and histogram plots (separated into quartiles) of estimated population densities (A), Housing Units (B) and (C) median income level of patients. The map shows the known locations of coronavirus subjects by residence zip codes. Red circles are sized by the number of study patients who resided in the region displayed. Adapted from the U.S. Census Bureau. American Community Survey, 2018 5-year estimates. Prepared by Cubit Planning, Inc. April 2020

The most common symptoms at initial presentation were dyspnea 39% (n=162) followed by cough 38% (n=156) and fever 34% (n=143). Other symptoms included myalgia, fatigue, nausea, and vomiting which occurred four to seven days prior to presentation. (Fig. 1) Self-reported exposure was confirmed in 26% of the patients. Figure 2 show the population densities (IA), number of housing units (IB) and median income levels (IC) of study participants compared to the rest of New Jersey. Minority patients admitted for COVID-19 were in the highest quartile for population’s density, number of housing units and disproportionately fell into the lowest median income quartile.

Shown in Table 1 is a comparison of baseline demographic, clinical and treatment characteristics between survivors and non-survivors. Overall, the study cohort had a median age of 62, and an interquartile range (49–74) years, were predominantly male with a 70% incidence of hypertension. More than half of patients had ground-glass opacities on chest imaging and most of them were treated with either hydroxychloroquine or antibiotics. Only two patients out of the whole cohort had received the anti-viral drug remdesivir as part of a clinical trial.

**Table 1:** Baseline demographic, clinical and treatment characteristics of patient population

Variable	Overall (n = 251)	Survivors (n = 154)	Non-survivors (n = 97)	p value
Age (years) (m, IQR)	62 (49–74)	58 (46–69)	68 (56–77)	< 0.0001
Sex (Male)	129 (51)	69 (45)	60 (62)	0.008
Race				
African American	210 (84)	122 (79)	88 (91)	0.013
Hispanic	41 (16)	32 (21)	9 (9)	
BMI (kg/m^2^)				
< 25	48 (19)	27 (18)	21 (22)	0.445
25–29.9	83 (33)	48 (31)	35 (36)	
30–35	61 (24)	38 (25)	23 (24)	
> 35	59 (24)	41 (27)	18 (19)	
Insurance Status				
Commercial	113 (45)	67 (44)	46 (47)	0.655
Medicare/Medicaid	86 (35)	52 (34)	34 (35)	
Charity Care/Uninsured	51 (20)	34 (22)	17 (18)	
Self-reported exposure				
Yes	65 (26)	39 (25)	26 (27)	0.795
History of				
Hypertension	175 (70)	98 (66)	77 (80)	0.016
Diabetes Mellitus	115 (46)	65 (45)	50 (52)	0.269
CAD	49 (20)	20 (14)	29 (30)	0.003
CHF	50 (20)	25 (17)	25 (26)	0.095
CVA	28 (11)	12 (9)	16 (16)	0.056
COPD	23 (9)	13 (9)	10 (10)	0.754
HIV	13 (5)	12 (8)	1 (1)	0.008
CKD	46 (18)	17 (12)	29 (30)	0.005
Malignancy	22 (9)	8 (6)	14 (14)	0.021
Duration of Symptoms (d)	4 (2–7)	4 (3–7)	3 (2–7)	0.189
Vital signs at the time of presentation				
Fever (> 100.4F)	143 (57)	89 (58)	54 (56)	0.741
HR > 125 bpm	16 (6)	6 (6)	10 (6)	0.922
RR > 24 bpm	55 (22)	28 (29)	27 (18)	0.036
MAP < 65	9 (4)	3 (3)	6 (4)	0.922
Chest Imaging features				
Consolidation	48 (19)	27 (22)	21 (22)	0.509
Ground glass Opacities	136 (54)	87 (56)	49 (51)	0.385
Bilateral Infiltrates	192 (76)	122 (79)	70 (73)	0.271
Treatment				
Chloroquine	186 (74)	102 (72)	84 (92)	0.001
Remdesivir	2 (1)	2 (2)	0 (0)	0.157
Steroids	35 (14)	9 (6)	26 (28)	< 0.001
Tociluzumab	39 (16)	16 (12)	23 (28)	0.002
*Antibiotics	204 (81)	118 (77)	86 (91)	0.004

M: Median; IQR: Interquartile range; CAD: Coronary artery disease; CHF: Congestive Heart Failure; CVA: Cerebrovascular Accident; COPD: Chronic Obstructive Lung Disease; HIV: Human Immune Deficiency virus; CKD: Chronic Kidney Disease; d: days; HR: Heart Rate; bpm: beats/breaths per minute; RR: Respiratory Rate; MAP: Mean Artery Pressure; *Anti-bacterial medications used in this category included azithromycin, ceftriaxone and doxycycline

Compared to survivors, non-survivors were significantly older, more likely to be male, with a higher incidence of hypertension, coronary artery disease and chronic kidney disease. In contrast, the incidence of Human Immunodeficiency Virus (HIV) infection was significantly higher among survivors than non-survivors (p = 0.008).

**Fig. 3:**
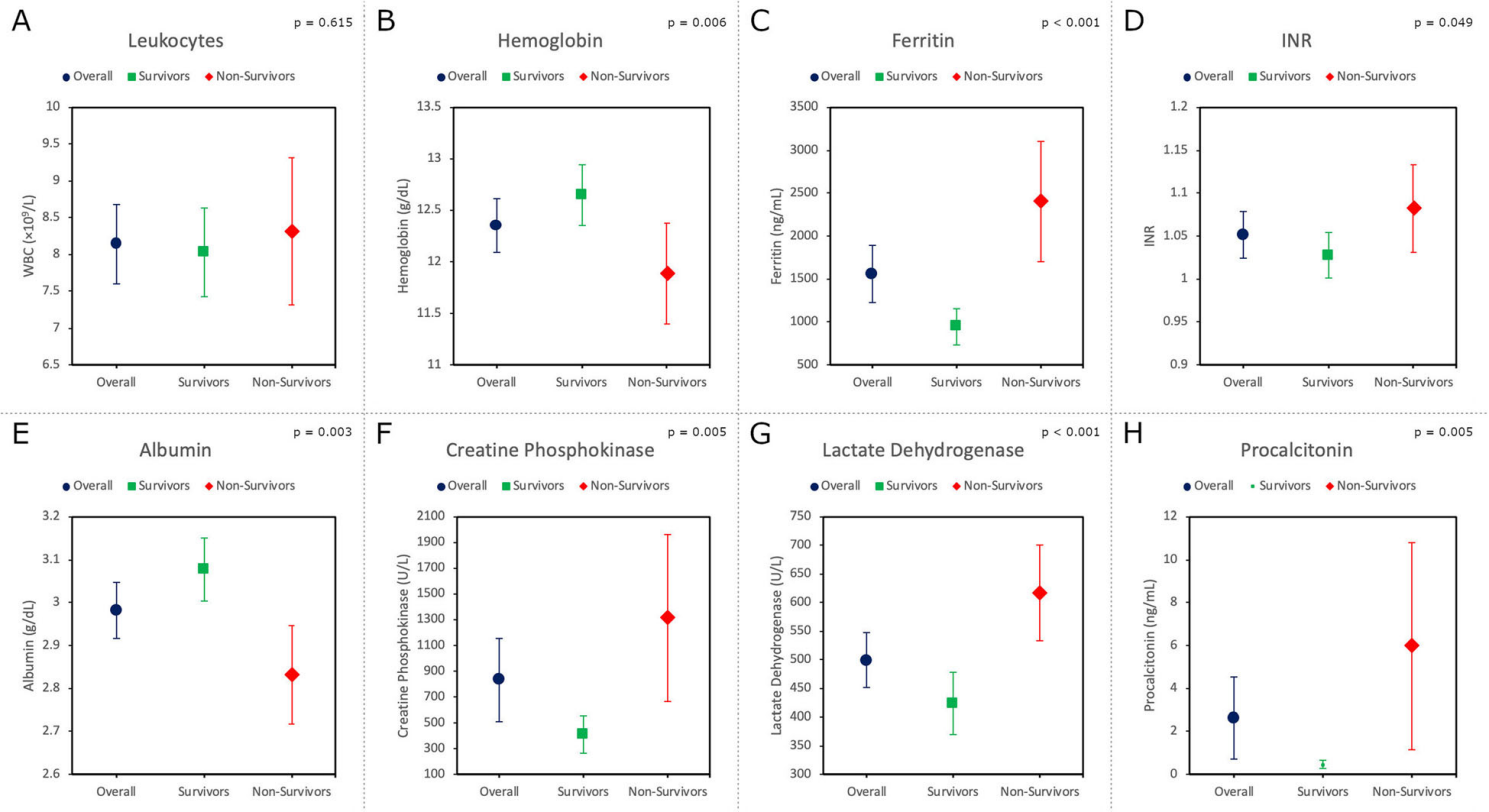
Schematic representation of laboratory values including serum white cell count (A), Hemoglobin (B), Ferritin (C), International Normalized Ratio (D), albumin (E), creatinine phosphokinase (F), Lactate Dehydrogenase (G) and Procalcitonin (H) compared between survivors and non-survivors

We tracked major laboratory values at baseline and compared their levels among survivors and non-survivors. Figure 3 (A-H) is a schematic representation of individual laboratory values of variables previously described in the literature. Significantly higher levels of serum Ferritin (p <  0.001), INR (p = 0.049), Creatinine Phosphokinase (p = 0.005), Lactate dehydrogenase (p <  0.001) and Procalcitonin (p = 0.005) were seen in non-survivors than survivors. Moreover, non-survivors had lower levels of serum Hemoglobin (p = 0.006) and albumin (p = 0.003) than survivors.

The overall incidence of ARDS, Septic shock, acute kidney injury requiring hemodialysis and admission to an intensive care unit (ICU) for worsening clinical status was 33% (n = 82), 24% (n = 59), 21% (n = 52), 33% (n = 82) respectively. Of the 82 patients who required ICU admission, 70 (85%) had expired at the time of last censor (April 20, 2020) and only 11% (n = 6) of patients who required dialysis for AKI survived to discharge.

After univariate analysis, the odds of in-hospital mortality were found to be higher in older patients, males, Black/African Americans, and those with hypertension, coronary artery disease and chronic kidney disease. (Table 2) The presence of HIV infection was associated with a survival benefit. Elevated serum Ferritin, LDH, PCT, CPK, Troponin levels but lower levels of serum albumin and Hb at baseline were also associated with mortality. In multivariable logistic regression analysis, independent predictors of in-hospital mortality were older age, HIV status, serum Hb > 10 mg/dl, Ferritin > 1200 ng/ml and CPK > 1000 U/L.

**Table 2:** Univariate and Multivariable logistic regression analysis of factors associated with in-hospital mortality

	Univariate Analysis		Multivariable Analysis	
Variable	OR (95% C.I)	p value	OR (95% C.I)	p value
Age (/unit increase)	1.04 (1.02,1.06)	< 0.001	1.04 (1.01, 1.06)	0.003
Sex (Male vs Female)	1.99 (1.19, 3.37)	0.002	–	–
Race (AA vs Hispanic)	2.56 (1.20, 5.95)	0.013	–	–
History of				
Hypertension	2.21 (1.23, 4.04)	0.007	–	–
CAD	2.85 (1.52, 5.48)	0.001	–	–
HIV	0.12 (0.06, 0.64)	0.006	0.07 (0.03,0.52)	0.006
CKD	3.43 (1.78, 6.80)	0.002	–	–
Baseline serum				
Hemoglobin (> 10 mg/dl)	0.22 (0.09, 0.47)	0.002	0.26 (0.07, 0.78)	0.016
Ferritin (> 1200 ng/L)	4.26 (2.37, 7.82)	< 0.001	2.91 (1.40, 6.17)	0.004
LDH (> 500 u/l)	2.47 (1.45, 4.26)	0.009	–	–
Procalcitonin (> 0.5)	4.32 (2.43, 7.82)	< 0.001	–	–
CPK (>1000u/l)	3.51 (1.55, 8.58)	0.002	3.04 (1.04, 9.44)	0.041
Troponin (> 0.1)	3.44 (1.56, 7.97)	0.002	–	–
Albumin (> 2.5 g/dl)	0.35 (0.16, 0.74)	0.006	–	–

OR: Odds ratio; C.I: Confidence Interval. CAD: Coronary artery disease; HIV: Human Immune Deficiency Virus; CKD: Chronic Kidney Disease; LDH: Lactate dehydrogenase; CPK: Creatinine phosphokinase

## Discussion

To our knowledge, this is the first study to describe in detail clinical outcomes of minority patients hospitalized for COVID-19 in an inter-city hospital. We found an in-hospital death incidence of 38.6% among Black/African American and Latino/Hispanic patients. These patients resided in areas of New Jersey with reportedly higher population densities, housing units and lower income levels than the rest of the state. Minority patients were more likely to present later in the course of disease with worsening shortness of breath and cough while being older, with a high serum ferritin, and creatinine phosphokinase levels, were associated with in-hospital death.

Earlier reports on outcomes after COVID-19 have highlighted the association between modifiable risk factors such as hypertension; diabetes and obesity with worse outcomes [10–12]. Recent data, [5, 6] from state healthcare departments have shown that, health and health care disparities (HHD) might be associated with worsened outcomes. While hand hygiene, social distancing, or physical isolation have been successfully implemented to flatten the curve in several parts of the world, the effects of HHD are more challenging to address. For instance, in cities like Chicago and Louisiana more than two-thirds of COVID-19 deaths occurred in black individuals although, blacks consisted of only a third of the population [5, 6]. According to published data on initial experience with COVID-19; in-hospital mortality has ranged between 0 to 27% [3, 13–15]. We found a disproportionately higher rate of 38.6% among the patients included in this study. This higher mortality may be explained by a higher burden of comorbidities like hypertension, diabetes mellitus that was seen in more than 60% of both the Black/African American and Latino-Hispanic patient population. Our study population resided in densely populated regions of New Jersey with higher number of housing units. The location of their residencies also correlated with a lower annual median income than most parts of New Jersey. As a result, underrepresented minorities including Blacks and Hispanics may endure the most of HHD since they do not have the luxury of social distancing or physical isolation in the era of a pandemic.

The most common presenting symptom in our cohort was dyspnea followed by cough then fever. About 43% patients presented with dyspnea. Zhou et al., in their outline of the clinical course of major symptoms for COVID-19 infected patients, identified dyspnea as one of the common symptoms occurring on day 6 of the disease course [3]. Fever on the other hand was an initial presenting symptom while cough was concurrent with fever. Our findings can be explained by a late presentation in the disease phase, which may be associated with a lack of desire to seek medical attention immediately after becoming symptomatic. Furthermore, this lack of desire may not be intrinsic but also socioeconomic related as most of these patients depend on a paycheck-to-paycheck budget to afford a living. A sign of dyspnea in this respect, may suggest deterioration in respiratory status because of alveolar damage or filling with fluids. Additionally, we noted significantly higher baseline serum levels of ferritin, albumin and procalcitonin in non-survivors than survivors. These markers have been reported as indicators of disease severity and further explains a late course in the disease process at the time of presentation [16].

The mortality and morbidity associated with COVID-19 is reportedly high according to findings from previous studies [3, 11, 13, 14]. In this case series, we found an overall incidence of admission to an intensive care unit (ICU) for worsening clinical status to be 33% (n = 82) with only 12 of these patients surviving till discharge. This finding is like what has been described in the literature to date [11], but interestingly does not correlate with the rate of in-hospital mortality of 38.6%. The higher in-hospital mortality in this series explains a higher rate of death within hours of presentation to our healthcare facility. In fact, a considerable number of patients who had died within hours of presentation did not make it to the intensive care unit. Moreover, patients who were deemed clinically stable on the general medical floors had experienced a rapid deterioration in their clinical status, leading to death without admission to the intensive care unit.

We identified three laboratory markers (namely➔hyperferritinemia, low serum hb and high level of CPK and older age, as independent predictors of in-hospital death. Similar to what was described by Ruan et al., [12] we found high levels of plasma ferritin to be significantly associated with in-hospital death. Another predictor of in-hospital death was a low serum hemoglobin. While some studies have postulated the direct impact of the viral protein on hemoglobin via immune hemolysis of red blood cells, [16] a low Hemoglobin could indicate signs of chronic disease among those with pre-existing conditions or may serve as a surrogate marker for active hemolysis especially in the Black/African American population. CPK levels were higher, in non-survivors than survivors and was an independent predictor of mortality. This finding may represent the impact of disease on muscle mass in the Black/African American population who are known to have significantly higher muscle mass than other ethnic groups [17].

Interestingly, we found that, the presence of HIV infection was associated with survival in this patient population. This might be an important consideration in the understanding the pathophysiology of the disease process. First, despite an extremely high prevalence of HIV in the region where our center is located, only 5% of patients who required admission for COVID had HIV. Second, it was surprising to note that, all HIV patients survived until discharge except for one patient, aged 70-years who died in-hospital due to multi-organ failure. Most of the patients who survived were on anti-retroviral medications with normal CD4 counts and well controlled viral loads. In a Spanish experience with HIV and COVID-19, the authors treated five patients with both anti-SARS-COV-2 treatment on day of diagnosis followed by boosted-protease inhibitor anti-retroviral therapy. Concomitant antibacterial were given to three patients who had pneumonia, corticosteroids in other two and tocilizumab in one patient. The authors highlighted a discharge rate of 80% at the time of their writing [18]. In fact, while the exact reason behind a survival advantage in these patients is unclear, the role of anti-retroviral drugs in the management of HIV patients who are co-infected with COVID will clearly pose a clinical challenge in the near future.

Our study has several limitations; first, this study is a retrospective study and is by all factors inherent to retrospective analysis. Second, the study does not include all laboratory variables previously described in the literature including interleukin levels and other variables needed to calculate risk assessment scores like SOFA scores at baseline. Third, although the patient population is predominantly Black/African American or Latino Hispanics, our results are limited by a single centers experience that may not be generalizable to the overall population. The exclusion of non-Latino/Hispanic patients from this study also limits the generalizability of the study findings. Our hospital population is mainly Black/AA or Hispanic patients, and there were only 10 non-Latino/Hispanic patients out of the who met the inclusion criteria. Fourth, due to the novel nature of COVID-19, there was no standard management protocol employed for all patients who were admitted. This would have likely contributed to the worse outcomes observed. Lastly, interpretation of our data should be done with caution due to the smaller sample size and lack of full knowledge on this disease.

## Conclusion

In conclusion, we present a single center’s experience with COVID-19 among underserved minority populations. These patients lived in densely populated areas with significantly lower median income and reported a higher in-hospital mortality rate than what is described in the literature. The incidence of in-hospital death was 38.6% and morbidity reported was like what has been described in the literature.

## Data Availability

All data will be available upon request.
